# Carotid Artery Stenting by Direct Punctures of Cervical Carotid Artery Stenoses Using an Exoscope in a Hybrid Operating Room: A Technical Report

**DOI:** 10.7759/cureus.70410

**Published:** 2024-09-28

**Authors:** Takeshi Miyata, Taketo Hatano, Takenori Ogura, Yuji Agawa, Yusuke Nakazawa

**Affiliations:** 1 Neurosurgery, Kokura Memorial Hospital, Kitakyushu, JPN; 2 Neurology, Kokura Memorial Hospital, Kitakyushu, JPN

**Keywords:** carotid artery stenosis, carotid artery stenting, direct puncture, exoscope, hybrid operating room

## Abstract

Carotid artery stenting (CAS) has been established as an effective surgical treatment for internal carotid artery stenosis and/or common carotid artery stenosis (ICAS/CCAS). Typically, CAS is performed via a transfemoral, transbrachial, or transradial approach. However, direct puncture CAS (DP-CAS) is preferred in cases where conventional access routes are challenging, such as in the presence of cervical vascular tortuosity or thoracic aortic aneurysm. Since 2020, our institution has selectively adopted DP-CAS, utilizing 3D exoscopy through the ZEISS KINEVO 900 microscope (Carl Zeiss AG, Oberkochen, Germany) or the ORBEYE exoscope (Sony Olympus Medical Solutions Inc., Tokyo, Japan) for carotid artery surgical exposure and puncture under high magnification in a hybrid operating room. In the hybrid operating room, exoscopic and angiographic fluoroscopic monitors were positioned side by side, enabling the surgeon to simultaneously visualize both during the puncture procedure. In cases utilizing the ORBEYE exoscope, its compact design minimized interference with the fluoroscopic arm, thereby facilitating improved working angles during CAS. This technical report reveals the current status of DP-CAS at our institution and focuses on the effectiveness of DP-CAS using an exoscope based on our clinical experience.

## Introduction

Carotid artery stenosis is a prevalent cause of ischemic stroke, accounting for approximately 10-20% of cases [1]. Carotid endarterectomy (CEA) has traditionally been performed for internal carotid artery stenosis and/or common carotid artery stenosis (ICAS/CCAS) [2,3]. In recent years, large clinical studies have demonstrated the efficacy of carotid artery stenting (CAS) in normal-risk patients with normal procedural risk when compared to CEA [3-5]. However, a combined treatment approach involving CEA and CAS, known as direct puncture CAS (DP-CAS), has been shown to be beneficial for ICAS/CCAS with combined procedural risk [6-12], such as difficult vascular access route and the atherosclerotic plaque located at high cervical bifurcation. Despite its advantages, DP-CAS poses challenges due to the routine use of antiplatelet therapy during revascularization, necessitating meticulous attention to perioperative complications related to the puncture site [13]. In our hybrid operating room, we perform DP-CAS through a small cervical incision, utilizing an exoscope under high magnification to ensure safe and accurate anterior wall puncture of the vessel, as well as secure hemostasis.

The introduction of exoscopes in neurosurgery has provided several benefits, including heads-up surgery, freedom from eyepiece constraints, a compact design, and three-dimensional (3D) 4K resolution visualization [14-16]. The exoscope's capability to offer an unrestricted visual axis has been reported as a significant advantage [14-16].

Our department introduced the ORBEYE exoscope (Sony Olympus Medical Solutions Inc., Tokyo, Japan) in September 2021, and it has since been employed in all scheduled micro-neurosurgical procedures. This technical note outlines our procedural experience with DP-CAS utilizing the exoscope for ICAS/CCAS patients with complex procedural risks, focusing on the safety and efficacy of direct puncture of the cervical internal carotid artery and/or common carotid artery (ICA/CCA) under high magnification in the hybrid operating room. The present report also highlights the effectiveness of utilizing the ORBEYE exoscope with its compactness and its free movability in preventing possible complications of DP-CAS.

## Technical report

Patient selection

The decision to treat ICAS/CCAS in our institution is based on a comprehensive pre-treatment evaluation of the following factors: (1) whether the ICAS/CCAS lesion is symptomatic, asymptomatic, or progressing; (2) whether the patient is refractory to maximum medical therapy; (3) age, activities of daily living (ADL), and medical history; (4) the position of the carotid bifurcation, and any anatomical variations; (5) the presence of severe calcification; (6) plaque instability; (7) evidence of cerebral hemodynamic ischemia, and the development of collateral circulation via Willis’ circle or leptomeningeal anastomosis; and (8) the vascular access route for endovascular treatment. Based on these factors, CAS is generally selected for cases that can be managed easily through endovascular intervention, while CEA is reserved for high-risk CAS cases, such as those involving unstable plaques. Furthermore, DP-CAS is selectively performed in cases where conventional vascular access for CAS (e.g., transfemoral or transradial approaches) is challenging, particularly in patients with abdominal or thoracic aortic aneurysms. DP-CAS is also considered for cases where stable plaque extends beyond the upper margin of the C2 vertebra or when symptomatic cervical spondylosis makes it difficult to secure optimal head positioning for CEA.

Operative technique

**Figure 1:**
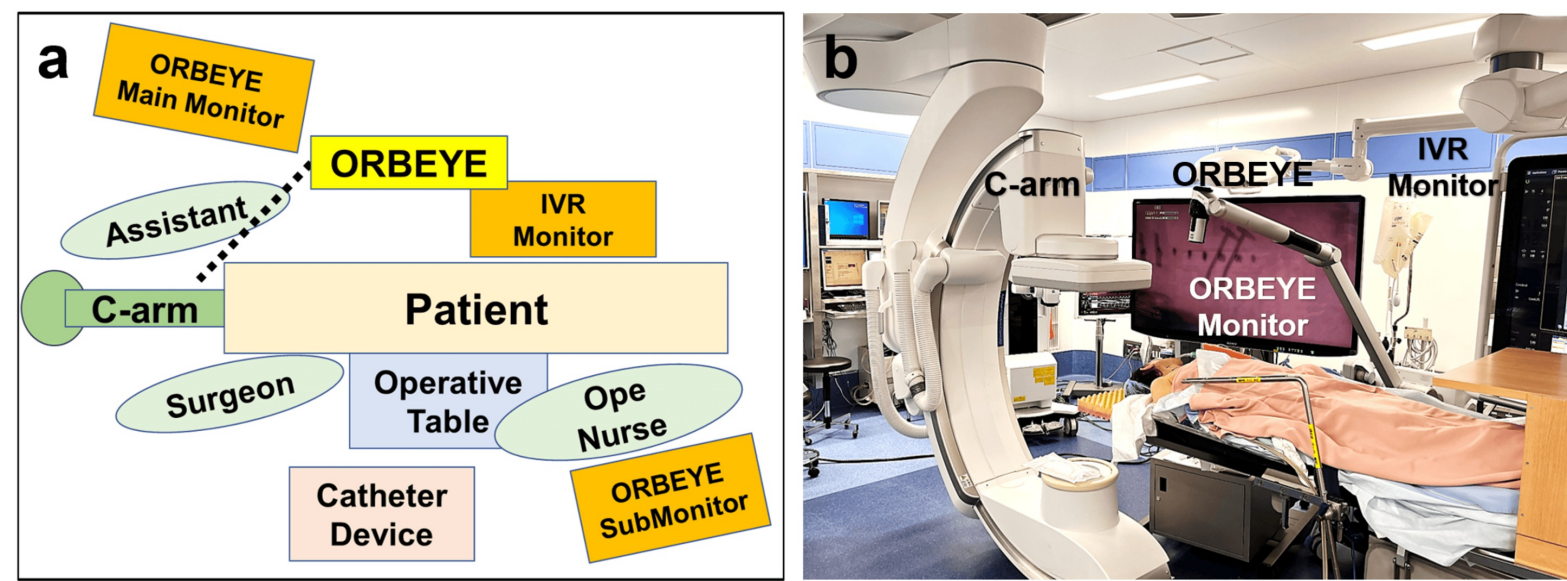
Setup in the hybrid operating room a) A schematic diagram showing the arrangement of the patient, C-arm, exoscope housing, exoscope monitor, and angiography monitor in the hybrid operating room. b) A photograph of the actual setup, demonstrating the extension of the exoscope arm between the C-arms to prevent interference with C-arm movements. ORBEYE exoscope (Sony Olympus Medical Solutions Inc., Tokyo, Japan)

Patients receive dual antiplatelet therapy for at least two weeks prior to surgery. In the hybrid operating room equipped with a robotic Flex C-arm system (Azurion, Philips Healthcare, Amsterdam, the Netherlands), the ORBEYE exoscope is positioned on the left side of the patient to avoid interference with the fluoroscopic C-arm and the ORBEYE exoscope monitor (Figure 1a, 1b). The ORBEYE exoscope monitor is arranged alongside the cerebral angiography monitor, allowing the surgeon to view both the operative field and angiographic images simultaneously. Under general anesthesia, the patient is placed in the surgical position with the head rotated approximately 45 degrees and the neck slightly extended. Intravenous digital subtraction angiography (IV-DSA) is performed by injecting a contrast medium via a venous route [17], generating a roadmap of the CCA to confirm its course and determine the skin incision and puncture site.

The anterior margin of the sternocleidomastoid muscle is identified, and the CCA is exposed and secured with a vascular tape under strong magnification via the ORBEYE exoscope through the oblique or transverse skin incision. Tobacco sutures are preplaced with Gore-Tex CV-7 sutures (Daishin Boeki, Osaka, Japan) around the puncture site for immediate hemostasis upon sheath removal. Heparin (3000-4000 units) is administered, and the CCA is punctured using an 18-gauge needle while being suspended by the vascular tape, taking care to avoid penetration of the posterior wall. A guidewire is advanced into the external carotid artery (ECA) under roadmap guidance with contrast media from the outer sheath of the puncture needle. A 6 Fr, 11 cm super sheath with a tip marker (Medikit, Tokyo, Japan) is inserted and fixed subcutaneously. In order to perform CAS, we ensure the establishment of an appropriate working angle that allows for optimal visualization of the stenotic region.

The following CAS is performed according to standard procedure. The proximal CCA is directly clamped using forceps from the surgical field for the proximal flow control, and a 6 Fr sheath is connected to the venous system to establish flow reversal. A distal filter protection system, such as a Spider FX (Covidien, Mansfield, Massachusetts, United States), is deployed beyond the stenotic lesion, followed by pre-dilatation with a percutaneous transluminal angioplasty balloon, stent placement, and post-dilatation to complete the CAS procedure. During sheath removal, under high magnification, both the angiographic and ORBEYE exoscopic monitors are used by the surgeon and the assistant to carefully achieve the ligation of the puncture site with the pre-applied sutures and effective bleeding control. Hemostasis is confirmed, and the wound is closed to complete the procedure.

Representative cases

Case One

**Figure 2:**
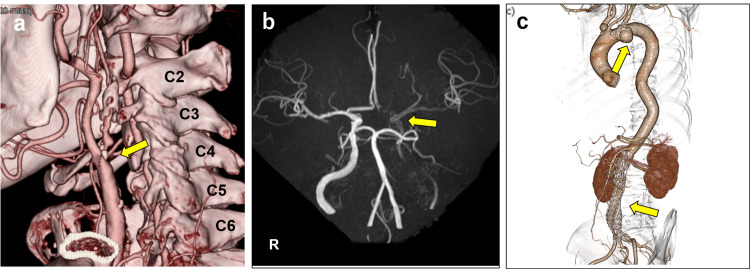
Asymptomatic severe stenosis of the left internal carotid artery (case one) a) Cervical contrast-enhanced 3D-CT angiography revealing severe stenosis of the left internal carotid artery (arrow). b) Head magnetic resonance angiography (MRA) showing poor visualization of the intracranial carotid artery and middle cerebral artery (arrow). c) Thoracoabdominal contrast-enhanced 3D-CT angiography demonstrating thoracic and abdominal aortic aneurysms post-stent graft placement (arrows)​.

A 62-year-old male had a history of abdominal aortic aneurysm and had previously undergone stent graft insertion for this condition. He also had a thoracic aortic aneurysm. Although the patient was asymptomatic, severe stenosis was detected in the left ICA (Figure 2a). Carotid ultrasonography revealed a peak systolic velocity (PSV) of 470 cm/sec at the stenotic site, indicating decreased blood flow. The plaque characteristics did not suggest instability. Given that the head magnetic resonance angiography (MRA) showed a pseudo-occlusion (Figure 2b), it was considered that revascularization was necessary. Based on the patient’s medical history (Figure 2c) and a long segment of ICAS/CCAS extending from the upper border of the second cervical vertebra to the fourth vertebra (Figure 2a), DP-CAS with exoscope was decided to be performed in the hybrid operating room.

**Figure 3:**
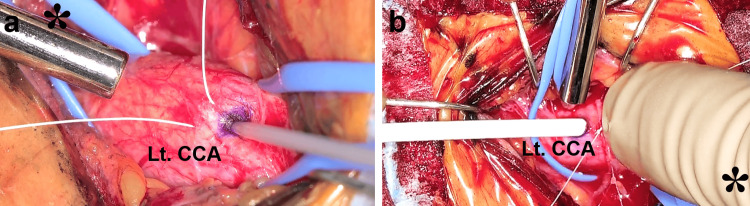
Intraoperative findings (case one) a) Under high magnification using the exoscope, the left common carotid artery (CCA) is punctured safely and precisely. b) Under high magnification using the exoscope, the left CCA is meticulously managed for hemostasis by the surgical team​. Asterisks indicate the side of the patient's head.

During the procedure, as described above, both the exoscopic monitor and the cerebral angiographic monitor were positioned within the surgeon’s field of view (Figure 1). During the direct cervical puncture, the surgeon and the assistant simultaneously monitored both the exoscopic and angiographic displays, ensuring meticulous avoidance of posterior wall puncture and vessel dissection (Figure 3a, 3b).

**Figure 4:**
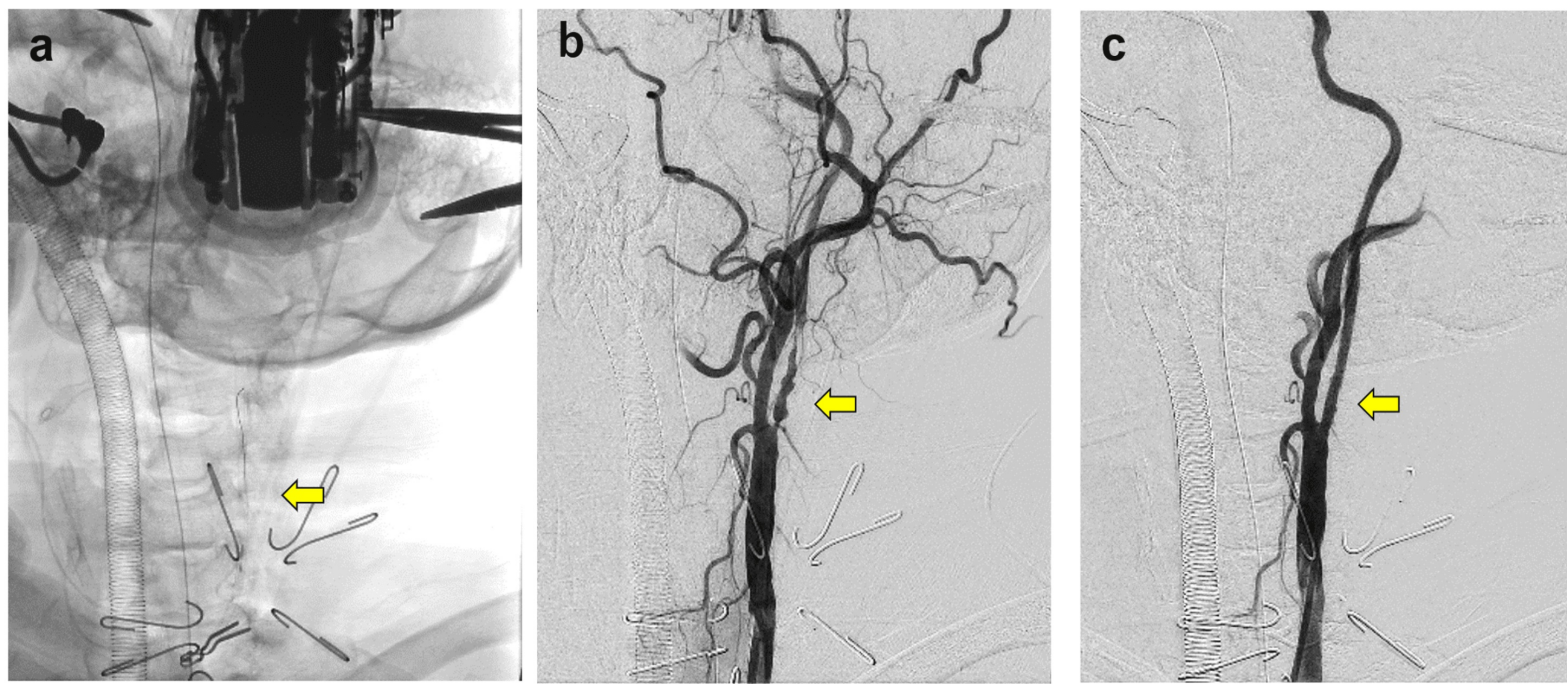
Carotid artery stenting in the hybrid operating room (case one) a) The placement of the sheath (arrow) punctured into the surgically exposed left common carotid artery (CCA). b) Digital subtraction angiography (DSA) of the left CCA confirming severe stenosis of the left internal carotid artery (ICA) (arrow) and secure placement of the sheath tip. c) DSA showing successful revascularization following carotid artery stenting (arrow).

The patient underwent CAS as per standard protocol, and successful revascularization was confirmed (Figure 4a, 4b, 4c). At the moment the sheath was removed, the tobacco sutures were carefully ligated with the surgeon and the assistant monitoring both exoscopic and angiographic displays' images under high magnification (Figure 3b). The postoperative course was uneventful, and the patient was discharged eight days later without any neurological deficits. During the three-year postoperative follow-up period, no imaging evidence of recurrent stenosis was observed.

Case Two

**Figure 5:**
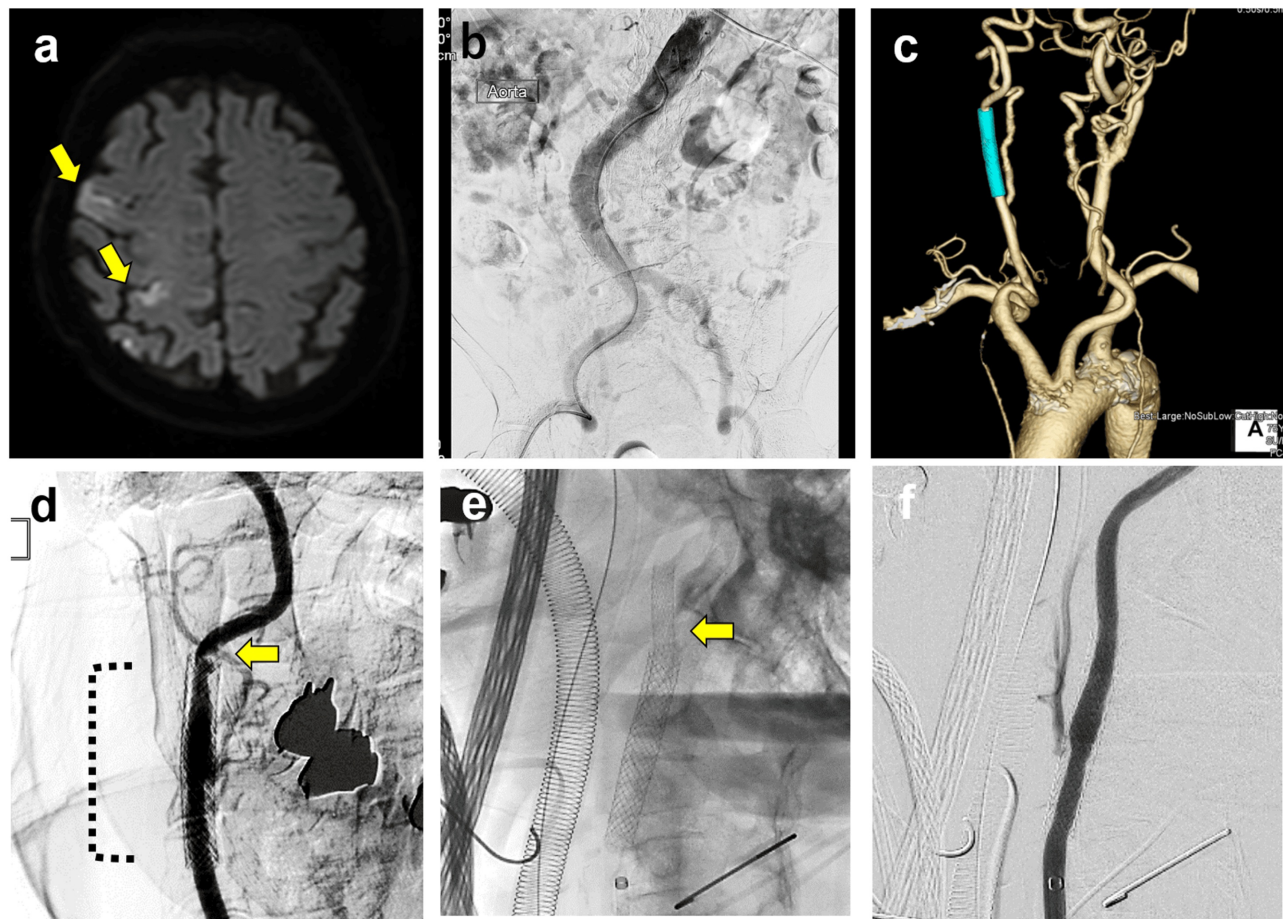
Symptomatic severe re-stenosis of the right internal carotid artery after the previous carotid artery stenting (case two) a) Head diffusion-weighted magnetic resonance imaging (MRI-DWI) demonstrates an acute cerebral infarction in the right frontal and parietal subcortical white matter (arrows). b) Digital subtraction angiography (DSA) reveals severe vascular tortuosity from the abdomen to both lower extremities. c) Cervicothoracic 3D-CT angiography demonstrating marked calcification of the thoracic aorta and significant tortuosity of the cervical vessels. d) DSA showing severe stenosis of the right internal carotid artery (ICA) (arrow), distal to the previously placed carotid stent (dotted line). e) Overlapping stent fully covering the stenotic lesion of the right ICA (arrow). f) DSA of the right common carotid artery confirming successful revascularization following carotid artery stenting.

At the age of 58, the patient underwent CAS for symptomatic right ICAS. The patient had a good course, but one year ago, restenosis at the stented area was observed. Carotid ultrasonography showed elevated blood flow with a PSV of 450 cm/sec at the stenotic region. There were no findings indicative of plaque instability. Subsequently, at the age of 76, the patient experienced an atherothrombotic stroke once again, with scattered infarcts observed in the right cerebral hemisphere (Figure 5a). After undergoing rehabilitation, the patient was scheduled for revascularization. Given the higher vessel tortuosity (Figure 5b, 5c), within the whole body, DP-CAS was performed in the hybrid operating room. The same procedure was performed and good revascularization was obtained (Figure 5d, 5e, 5f). The postoperative course was highly favorable, with no neurological abnormalities observed, and the patient was discharged home nine days after the procedure. Notably, no symptomatic postoperative complications or restenosis have been observed during the two-year follow-up period. 

## Discussion

We reported on DP-CAS utilizing an exoscope in a hybrid operating room for ICAS/CCAS with combined procedural risk. This report also demonstrated that the use of an exoscope during DP-CAS allowed for precise avoidance of vascular injury at the puncture site and ensured effective bleeding control during sheath removal, with high-magnification monitoring by the surgeon.

The standard of surgical treatments for ICAS/CCAS are CEA and CAS, and the choice between them should be based on the individual risk factors of each case [2-5]. Risk factors for CEA include medical conditions such as a history of severe cardiac or respiratory failure, as well as contralateral vocal cord paralysis. Anatomical considerations, such as high-positioned lesions or occlusion of the contralateral ICA, also pose risks [2,3]. In contrast, risk factors for CAS include medical conditions like contrast allergies and chronic kidney disease, alongside anatomical challenges such as difficult catheter access due to higher vascular tortuosity, unstable plaque, and heavily calcified plaque [4,5]. In some cases, both CEA and CAS carry high risks, and in such instances, the utility of DP-CAS has been reported [6-12]. In the present cases, we adopted DP-CAS due to the specific risk factors associated with both CEA and CAS. This approach resulted in successful recanalization without any complications.

DP-CAS has been reported to present challenges in achieving hemostasis following sheath removal [6,13]. Manual compression of the carotid artery can lead to reduced intracranial blood flow, hematoma formation due to insufficient compression, and respiratory distress from neck swelling. Local hematoma formation is a relatively common complication, with incidence rates reported as high as 7% [7,8,18]. Furthermore, DP-CAS requires reliable hemostasis, particularly because the procedure is performed under multiple antithrombotic therapies common in neuroendovascular treatments [6,13]. In this regard, DP-CAS with direct exposure of the carotid artery has been shown to provide more controlled hemostasis. In addition, the use of a hybrid operating room with a high level of cleanliness has been reported to minimize the risk of infection in such procedures [7]. However, accurately puncturing the anterior wall of the carotid artery, even under direct visualization, and achieving hemostatic control of the surrounding tissues can still be challenging [7,8,18]. Performing these procedures with high magnification can enhance the safety and precision of DP-CAS.

The exoscope has been developed as a novel surgical support system to replace the traditional operating microscope [14]. Since its inception, exoscope-assisted surgeries have been performed across various surgical specialties, with continuous innovations in the technology. ORBEYE, a leading representative of the current exoscope systems, offers 4K-3D high-resolution imaging on a large 55-inch screen, allowing for heads-up surgery, observation without the constraints of eyepiece limitations, and compact design [15,16]. These features have demonstrated significant utility in neurosurgery, as previously reported [15,16]. In September 2021, we introduced the exoscope ORBEYE in our institution, and we also introduced an exoscope for DP-CAS in the hybrid operating room. The exoscope mode of the Kinevo (Carl Zeiss AG, Oberkochen, Germany) operating microscope has been useful, but its large size and possible interference with the fluoroscopic arm have restricted its placement and limited the observation space. The ORBEYE has a small and compact body and a freely movable arm that allows unrestricted observation of the eyepiece, thus reducing interference with the fluoroscopic arm and enabling DP-CAS at more appropriate working angles for CAS. Recently, the usefulness of trans-carotid revascularization (T-CAR) for ICAS/CCAS has been reported mainly in the United States [19,20]. We have selectively performed DP-CAS for ICAS/CCAS with combined procedural risk with an exoscope as a therapeutic alternative since T-CAR has not been introduced yet in Japan.

## Conclusions

We have focused on the effectiveness of DP-CAS with an exoscope in a hybrid operating room, aimed at minimizing complication associated with puncture and/or hemostasis in ICAS/CCAS patients with complex treatment risks. In such cases, it is crucial to conduct a comprehensive preoperative assessment of all potential risk factors. Furthermore, careful coordination between the positioning of equipment and monitors is essential to ensure a safe and successful outcome.
